# Changes in the network structure of energy markets and financial markets under the different shocks of the Russia-Ukraine conflict and COVID-19

**DOI:** 10.1371/journal.pone.0318291

**Published:** 2025-04-07

**Authors:** Fuyao Li, Mu Tong, Shuhao Guan

**Affiliations:** 1 School of Finance, Southwestern University of Finance and Economics, Chengdu, China; 2 Collaborative Innovation Center of Financial Security, Southwestern University of Finance and Economics, Chengdu, China; 3 School of Chinese Financial Studies, Southwestern University of Finance and Economics, Chengdu, China; 4 School of Computer Science, University College Dublin, Dublin, Ireland; Shanghai Jiao Tong University, CHINA

## Abstract

Employing the quantile coherency method, we analyze the different impacts of the Russia-Ukraine conflict and the global shock caused by COVID-19 on energy and stock markets, highlighting how market behaviors varied under these two crises. The findings reveal that natural gas performed better than oil during the pandemic and the Russia-Ukraine conflict in the short and medium term. In contrast, oil outperforms natural gas over the long term.

## 1 Introduction

The global economy has faced unprecedented turbulence in recent years due to a series of intertwined crises spanning finance, health, geopolitics, and the environment. The onset of the COVID-19 pandemic in late 2019 led to widespread disruptions in human mobility and supply chains, severely affecting the supply and demand of goods and triggering significant downturns in international financial markets. For instance, the U.S. stock market experienced four circuit breakers [1] within eight consecutive trading days, reflecting heightened market volatility and uncertainty. While the global economy has not yet recovered from the impact of the pandemic, the Russian invasion of Ukraine in February 2022 introduced a new layer of geopolitical tension and economic uncertainty, marking one of the most severe conflicts in Europe since World War II. The imposition of unprecedented sanctions on Russia by many countries [2,3] further complicated the global economic landscape.

Russia’s pivotal role in global energy markets, particularly its significant exports of natural gas and oil to European countries, means that the conflict has had profound implications for energy supply chains. The war disrupted energy commodity trade and logistics, leading to surging prices that exacerbated pre-existing market instabilities caused by the pandemic. These disruptions not only influenced the traditional energy sectors but also impacted the renewable energy industry [4], as investors reassessed risks and opportunities within the energy portfolio. Additionally, the increasing costs of these commodities have the potential to affect global financial markets through financialization channels [5], thereby heightening tensions and uncertainty in the financial sector.

Simultaneously, the escalating risks associated with global warming and climate change [6–8] have direct implications for the energy market. Factors such as extreme weather events and rising temperatures are expected to affect energy supply and demand dynamics [9,10]. Climate-related risks, including natural disasters and policy shifts towards sustainable energy, can significantly influence financial markets by altering investor expectations and asset valuations. These multifaceted developments have intensified investors’ focus on the interplay between energy and financial markets.

Investor behavior plays a crucial role in financial market dynamics, and during periods of crisis, it becomes especially significant. Heightened uncertainty can lead to sharp fluctuations in asset prices across both energy and financial markets. Investors, as primary stakeholders [11,12] in these markets, directly influence market trends through their decisions and actions. Their risk preferences, demands, and market expectations contribute to market volatility and can exacerbate or mitigate the effects of economic shocks.

In times of market stress or financial turmoil, investors often engage in a ‘flight-to-safety’ strategy, selling off higher-risk assets in favor of lower-risk or ‘safe-haven’ assets, even at the expense of lower returns. Sudden events like financial crises or geopolitical conflicts can trigger this behavior, as investors seek to protect their portfolios from losses [13]. According to [14], a safe haven is defined as "an asset that is uncorrelated or negatively correlated with another asset or portfolio in times of market stress or turmoil" (p. 219). This means that during periods of market downturns, a safe-haven asset can provide protection by maintaining its value or appreciating, thereby offsetting losses in other investments.

While traditional safe-haven assets like gold have been extensively studied, the role of energy commodities as potential safe havens or hedging instruments has gained interest [15], particularly given the increasing financialization of energy markets. The interconnection between financial markets and energy commodities, such as oil and natural gas, suggests that these assets may offer diversification benefits or serve as hedges against financial market volatility. For example, oil has historically been considered for its hedging properties due to its fundamental role in the global economy. However, the performance of these energy commodities during recent crises, such as the COVID-19 pandemic and the Russia-Ukraine war, remains an area requiring further investigation.

These two events have imposed significant shocks on both financial and energy markets. The pandemic has disrupted global economic activity, leading to unprecedented volatility, while the Russia-Ukraine conflict has caused substantial fluctuations in energy prices, notably in natural gas, due to concerns over supply routes like the Nord Stream pipeline [16]. Consequently, understanding whether energy commodities can act as safe havens or effective hedges during such periods is of paramount interest to investors seeking to mitigate risks.

Some studies have begun to explore the safe-haven properties of various assets during the Russia-Ukraine conflict. for instance, [17] utilized an event study methodology to provide evidence of a flight from the ruble to currencies like the dollar and yen, as well as assets such as silver, brent, WTI, and natural gas in the immediate aftermath of the conflict. Similarly, [18] employed the wavelet coherence method to investigate the hedging properties of different asset classes against geopolitical risks during the conflict. However, there remains a gap in the literature regarding the comparative behavior of energy commodities as hedges or safe havens across different types of shocks.

To address this gap, our study employs the quantile coherency approach developed by [19], a method that has gained traction among scholars for analyzing nonlinear relationships between assets under varying market conditions [20–24]. Unlike traditional methods, such as the continuous wavelet transform [25], which illustrates dependence structures in the time-frequency domain but cannot capture interdependencies across different quantiles, the quantile coherency approach allows for a more nuanced analysis. It captures dynamics across various quantiles, including extreme tails, providing valuable insights into tail risks and the behavior of assets under different market stress levels. This is particularly important for investors formulating hedging and portfolio strategies across different investment horizons and market conditions. In addition, the hedging effects of these portfolios are further analyzed using the hedging efficiency and hedging ratios of [26].

Our research aims to investigate the correlation between energy commodities and global financial markets under varying shock scenarios, focusing on different quantile levels (0.05,0.5,0.95) over three distinct periods: the pre-COVID-19 era (representing normal market conditions), the COVID-19 pandemic era (characterized by widespread market turmoil), and the Russia-Ukraine war period (marked by supply chain disruptions). We analyze two essential energy commodities—natural gas and oil—and include gold and wheat for comparative purposes, given gold’s traditional role as a safe-haven asset and wheat’s relevance due to Russia’s significant export capacity. Additionally, we consider stock markets classified by MSCI into developed, emerging, and frontier markets, providing a comprehensive overview of global stock market performance based on economic development, size, liquidity, and market access criteria.

By examining the dependence structures at different frequencies and quantiles, our study contributes to the literature by providing a detailed understanding of how energy commodities interact with financial markets during different types of shocks. This research not only fills a gap in the existing literature but also offers practical implications for investors and policymakers regarding asset allocation, risk management, and the formulation of hedging strategies under varying market conditions.

The remainder of the paper proceeds as follows: Sect 2 reviews the relevant literature, Sect 3 outlines the data and methodology, Sect 4 presents the results and discussion, and Sect 5 offers concluding remarks.

## 2 Literature review

There is a rapidly growing literature focused on the impact of major shocks on financial markets. In response to recent major shocks, many studies have examined the effects of the COVID-19 epidemic on various financial market categories [27–29], which highlight significant market volatility and systemic risk increases. In addition, some papers have also studied global financial market fluctuations, such as those triggered by the collapse of Silicon Valley Bank [30–32], emphasizing the vulnerability of financial systems to both external health crises and internal financial shocks. Similarly, the Russia-Ukraine conflict disrupted supply chains, trade and logistics for energy commodities and then contaminated global financial markets through the channels of financialization[5], increasing uncertainty and volatility.

The COVID-19 pandemic and the Russia-Ukraine conflict have had significant and diverse impacts on global financial markets and the global economy. Many studies have examined these crises from various perspectives. At the corporate level, research consistently shows that firms with higher Environmental, Social, and Governance (ESG) ratings tend to perform better during the COVID-19 pandemic. For instance, [33] found that responsible companies in G20 countries benefited from their ESG strategies during the pandemic. Similarly, [34] demonstrated that investors shifted their preference toward firms with higher ESG scores and stronger creditworthiness, particularly in the fixed-income market. In contrast, the Russia-Ukraine conflict has prompted different responses in the corporate sector. Studies have focused on how companies’ decisions to remain or withdraw from the Russian market were influenced by ESG factors. For example, [35] found that firms with lower ESG scores were more likely to maintain operations in Russia, while those with higher ESG scores were more inclined to exit, facing less severe stock market reactions. Additionally, [36] analyzed market performance and found that firms that stayed in Russia underperformed both the market benchmark and firms that left. Moreover, [37] highlighted the superior performance of energy firms during the conflict.

Studies also focus on the level of global stock markets as the fear and uncertainty caused by the pandemic and the Russia-Ukraine conflict had a detrimental effect on stock market performance. [38] confirmed that fear arising from the severity of the pandemic led to rapid declines in global stock markets. Additionally, [39] observed that stock markets reacted swiftly to the pandemic, with market returns consistently falling in response to rising COVID-19 cases.In contrast, studies on the Russia-Ukraine conflict emphasise the distinct negative effects of geopolitical tensions on stock markets. In their study, [40] identified a significant negative relationship between the conflict and the global stock market. Likewise, [41] observed a negative impact of this military action on the G20 and other selected stock markets. Furthermore, [42] provided evidence of negative cumulative abnormal returns for global stock market indices, indicating heterogeneous effects during this invasion. Their findings reveal the vulnerability of globalized economies’ markets when facing international conflicts.

The existing literature has extensively examined financial assets’ interconnectedness during the COVID-19 pandemic and the Russia-Ukraine conflict. During the COVID-19 pandemic, several studies highlight the increased interconnectedness and heightened volatility in global markets. For instance, [43] found that the pandemic intensified risk spillovers between the US and Chinese stock markets. Similarly, [44] examined G20 stock markets and identified a significant increase in volatility connectedness during the crisis. [45] found that the pandemic had a significant impact on global financial markets. [46] employed the TVP-VAR model to study how the pandemic influenced the interconnectedness of commodity and financial markets, and they found evidence of strong volatility across the market, especially at the lower and middle-level quantiles. Similarly, [47] examined the dynamic relationships between commodities and stock prices during the pandemic, identifying a significant connection between gold prices with stock prices and oil prices for all Asian stock markets. During the Russia-Ukraine conflict, [48] measured the dynamic connectedness among Russia, European financial markets, and the global commodity markets using TVP-VAR approaches and reported that the relationship among the financial markets is more robust than before the conflict. Employing the same methodology, [49] estimated the connectedness of global commodity markets and reached a similar conclusion that the return spillover increased during the war. [50] also examined the interconnectedness between oil and other financial markets. Furthermore, [17] showed negative dynamic conditional correlations between the ruble and other assets, verifying the flight-to-safety phenomenon from the ruble to the USD, yen, silver, Brent, WTI and natural gas.

Energy economics has been a focal point in research, both during the COVID-19 epidemic and the Russian-Ukraine war [22,51–55]. During the COVID-19 pandemic, several studies explored the relationship between energy markets and stock markets. [56] found that energy market efficiency declined during the pandemic, while [57] showed that the number of COVID-19 cases in the US had both short- and long-term effects on crude oil and natural gas markets. Additionally, [58] identified negative returns for both oil and stock markets during the pandemic. [59] further examined the relationship between oil prices and US stock markets before and after the crisis and found that both upward and downward correlations between the two markets increased during the crisis. On the other hand, during the Russia-Ukraine conflict, some literature documents the performance of energy markets and firms during the war [37,50,60]. In particular, several studies have tried to uncover the link between energy and stock markets. [61] demonstrated the economic benefits of hedging stocks with oil using the DCC model. [62], employing a TVP-VAR method and a spillover index model confirmed the energy market’s significant role as a risk recipient from the stock market before COVID-19, with an increased acceptance of risk after the COVID-19 outbreak. [63] used the connectedness approach and QC Regression to study the correlation between fluctuations in oil prices and the returns of G7 stocks. The results prove that high oil prices tend to impact stock markets positively. [64] explored the dynamic co-movement between oil and six stock markets using two types of wavelet analysis. [65] investigated the impact of the Russian invasion of Ukraine on agricultural and energy commodities, highlighting significant heterogeneity and a pronounced effect on European natural gas markets.

In this paper, we conduct the quantile coherency approach proposed by [19], which has been widely utilized in recent literature [24,66,67], to investigate the differential impacts of the Russia-Ukraine conflict and the global shock caused by COVID-19 on energy and stock markets. This method captures nonlinear dependencies between assets across different frequency domains. Notably, no previous studies have applied this method to examine the relationship between energy and stock markets during the Russia-Ukraine conflict. By using this innovative approach, our study offers a unique contribution by exploring the role of energy commodities in global stock markets across three distinct periods. This research significantly advances the existing literature by providing new empirical evidence on the dynamic interactions between energy markets and international financial markets during periods of heightened uncertainty and volatility.

## 3 Data and methods

### 3.1 Quantile coherency approach for modelling energy and stock market networks

We employ the quantile coherency approach proposed by [19]. This method allows direct estimates of extreme co-movements between asset returns. It can capture specific dependencies in different parts of the distributions of both time series and at different frequencies, which improves accuracy across different market conditions. We define the dynamic dependence between two strictly stationary processes $x_{t,j1}$ and $x_{t,j2}$, which is called the quantile coherency kernel; the equation below is:


$$\begin{array}{ccc} R^{j_{1},j_{2}}(\omega ;\tau_{1},\tau_{2})=\frac{f^{j_{1},j_{2}}(\omega ;\tau_{1},\tau_{2})}{{\left(f^{j_{1},j_{1}}(\omega ;\tau_{1},\tau_{2})f^{j_{2},j_{2}}(\omega ;\tau_{1},\tau_{2})\right)}^{1∕2}} & & \end{array}$$ (1)

Where $j_{1},j_{2}\in \{1,\ldots ,d\},\omega \in \mathbb{R},\tau_{1},\tau_{2}\in [0,1],f^{j_{1},j_{2}}$ represents the quantile cross-spectral density $f^{j_{1},j_{1}}$ and $f^{j_{2},j_{2}}$ are the quantile spectral densities of processes $x_{t,j1}$ and $x_{t,j2}$, respectively and they are obtained from the Fourier transform of the quantile cross-covariance kernels’ matrix:


$$\begin{array}{ccc} \Gamma_{k}(\tau_{1},\tau_{2})={\left(\gamma_{k}^{j_{1}j_{2}}(\tau_{1},\tau_{2})\right)}_{j_{1},j_{2}=1,\ldots ,d} & & \end{array}$$ (2)

where


$$\begin{array}{ccc} \gamma_{k}^{j_{1}j_{2}}(\tau_{1},\tau_{2})=\text{Cov}(I\{X_{t+k,j_{1}}\leq q_{j_{1}}(\tau_{1}),X_{t,j_{2}}\leq q_{j_{2}}(\tau_{2})\}) & & \end{array}$$ (3)

*k* ∈ *ℤ*, *I* { *A* }  denotes the indicator function representing event *A*. When $j_{1}\neq j_{2}$ we can obtain cross-section dependence and by varying *k*, we can get important information about serial dependence. For the frequency domain, the matrix of quantile cross-spectral density kernels is defined as follows:


$$\begin{array}{ccc} f(\omega ;\tau_{1},\tau_{2})={\left(f^{j_{1}j_{2}}(\omega ;\tau_{1},\tau_{2})\right)}_{j_{1},j_{2}=1,\ldots ,d} & & \end{array}$$ (4)

where


$$\begin{array}{ccc} f^{j_{1}j_{2}}(\omega ;\tau_{1},\tau_{2})={\left(2\pi \right)}^{-1}\overset{\infty }{\underset{k=-\infty }{\sum }}\gamma_{k}^{j_{1}j_{2}}(\tau_{1},\tau_{2})e^{-ik\omega } & & \end{array}$$ (5)

To be specific, the quantile coherency is derived by the smoothed quantile cross-periodograms (see [19] for more details). In this study, we extract quantile coherency matrices for three quantiles (0.05,0.5, and 0.95). Also, we consider three frequencies: short-term (one week), mid-term (one month) and long-term (one year). Following [19], we use returns standardized by the conditional volatility estimated by a GARCH(1,1) model [68]. Then, we utilize the estimated quantile coherency as inputs for constructing the adjacency matrix to establish a tail-dependent network.

### 3.2 Hedging effectiveness

Furthermore, we estimate the hedging effectiveness of brent, gas, gold and wheat for the stock markets by employing the hedging effectiveness (HE) index [26], which reinforces the results of the quantile coherency approach from the perspective of the time domain.

Assume $R_{H,t}$ is the return on the hedged portfolio that combine a stock market index and an asset:


$$\begin{array}{ccc} R_{H,t}=R_{S,t}-\omega_{t}R_{A,t} & & \end{array}$$ (6)

where $R_{S,t}$ indicates the return of a stock market, $R_{A,t}$ denotes the return of an asset and $\omega_{t}$ is the hedge ratio. Hence, given an information set $I_{t-1}$, the hedged portfolio’s conditional variance can be expressed as follows:


$$\begin{array}{ccc} \text{var}(R_{H,t}I_{t-1})=\text{var}(R_{S,t}I_{t-1})-2\omega_{t}\text{cov}(R_{A,t},R_{S,t}I_{t-1})+\omega_{t}^{2}\text{var}(R_{A,t}I_{t-1}) & & \end{array}$$ (7)

Optimal hedging ratios (OHRs) are obtained when the conditional variance of the hedged portfolio is minimized:


$$\begin{array}{ccc} \omega_{t}^{*}I_{t-1}=\frac{\text{cov}(R_{S,t},R_{A,t}|I_{t-1})}{\text{var}(R_{A,t}|I_{t-1})} & & \end{array}$$ (8)

In order to obtain the OHRs, we apply the ADCC-GARCH model [69] (which can not only compute the time-varying correlation of multiple variables but also capture the asymmetry in conditional volatilities) to estimate the conditional volatility and covariance, and then compute the optimal hedge ratios. Accordingly, a long position in a given stock market index is hedged with a short position in brent(gas, gold or wheat) under the following specification:


$$\begin{array}{ccc} \omega_{t}^{*}|I_{t-1}=\frac{h_{SA,t}}{h_{A,t}} & & \end{array}$$ (9)

where $h_{SA,t}$ represents the conditional covariance and $h_{A,t}$ is the conditional variance of bitcoin (oil or gold) returns. Ultimately, the HE index by the equation below:


$$\begin{array}{ccc} HE=\frac{{\text{var}}_{\text{unhedged}}-{\text{var}}_{\text{hedged}}}{{\text{var}}_{\text{unhedged}}} & & \end{array}$$ (10)

According to [26], a higher HE index signifies higher hedging or diversification benefit.

### 3.3 Data

The data include two essential energy commodities (natural gas and oil), the three largest stock markets according to MSCI classification, supplemented by precious metals (gold) and grain (wheat), all denominated in US dollars. We use futures settlement prices for Brent North Sea crude at the International Petroleum Exchange (IPE) in London, natural gas, COMEX gold, and CBOT wheat at the New York Mercantile Exchange (NYMEX) in the U.S. And we employ the closing prices of the stock markets. MSCI classifies global markets into developed, emerging, and frontier markets based on the country’s economic development level, the degree of market access for foreign investors, and size and liquidity criteria from the perspective of investable stocks. Developed markets mainly include Canada, the United States, the United Kingdom, France, Austria, Finland, Japan, etc. Brazil, Chile, Hungary, Russia, China, the Philippines, etc., belong to the emerging market. Frontier markets cover Argentina, Croatia, Lithuania, Kenya, Mauritius, Bangladesh, etc. All the data are obtained from the “Wind” database (https://www.wind.com.cn). The sample spans from January 3, 2019, to October 6, 2022, and is divided into three periods: before the onset of COVID-19(January 3, 2019 to March 10, 2020), during the pandemic (March 11, 2020 to February 23, 2022), and during the Russia-Ukraine war (February 24, 2022 to October 6, 2022). We take the log difference in prices to obtain daily returns (in per cent).

**Fig 1:**
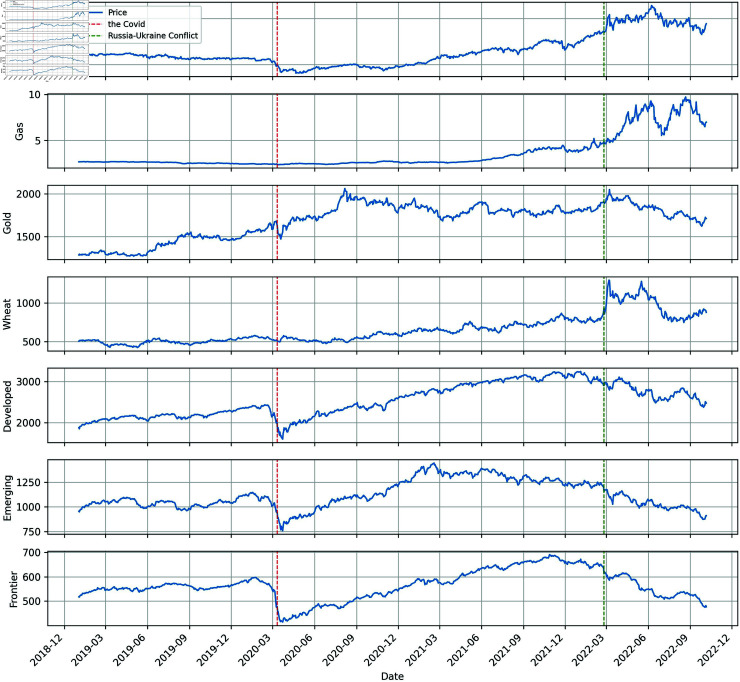
Time series plot of raw data.

Fig 1 shows the time series for each variable. We can see that around March 2020, there is a significant drop in the prices of all stock markets, which may be attributed to the World Health Organization’s declaration of the Coronavirus outbreak at that time. Meanwhile, the decline in oil and gold prices is similar to that of the stock markets, albeit slightly lesser.

**Fig 2:**
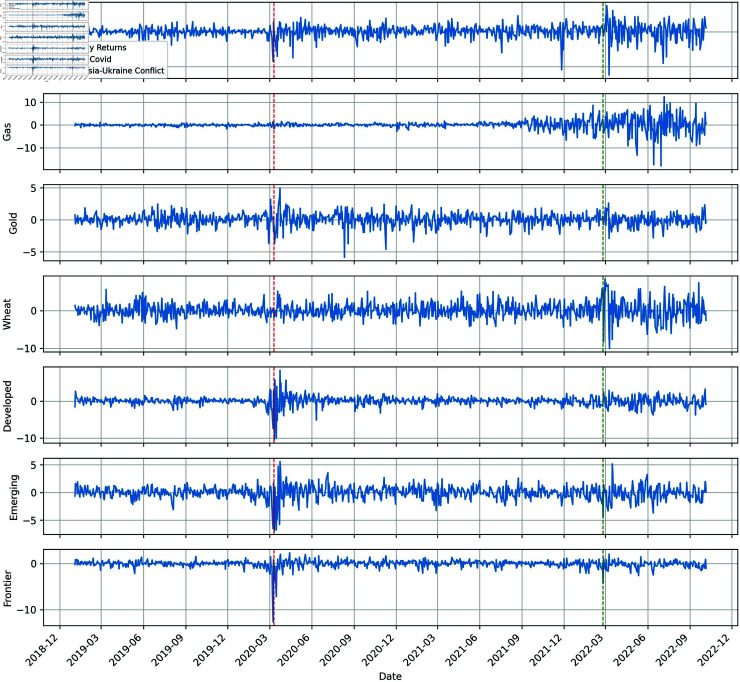
Dynamics of the sample returns.

Fig 2 plots the evolution of the price returns of the assets. The price returns exhibit significant volatility aggregation during COVID-19 and the Russia-Ukraine conflict, except for gas, which remains unaffected by COVID-19. These results suggest that the pandemic and conflict contribute to financial instability and considerably impact financial markets. All results also demonstrate evidence of nonlinear behaviour in price returns, indicating the feasibility of conducting studies using the quantile coherency approach.

**Fig 3:**
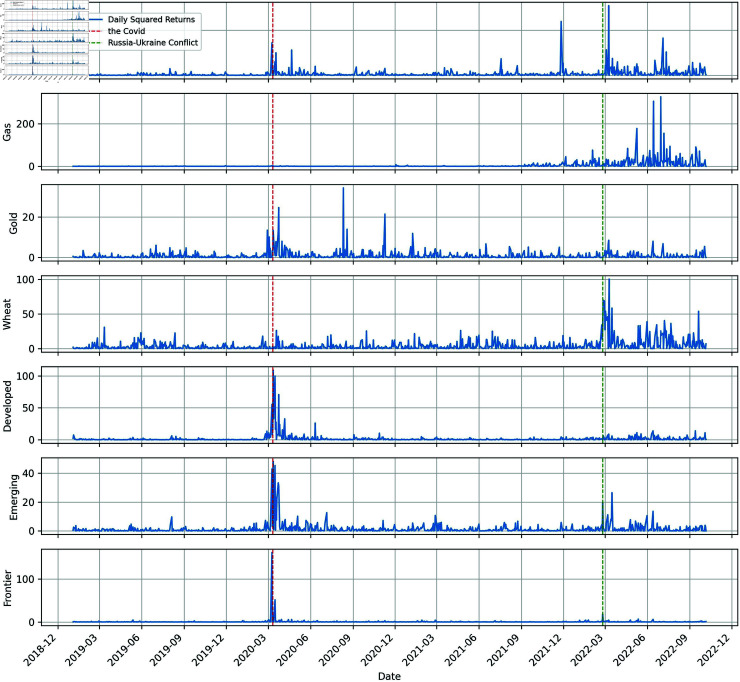
Dynamics of the sample squared returns.

Fig 3 displays a time series plot of squared returns, showing the volatility changes. Each sequence has a significant fluctuation aggregation effect around March 2020, except for natural gas and wheat. Oil and wheat exhibit apparent volatility immediately when the conflict begins, while the large fluctuations in the gas are somewhat delayed. And among stock markets, the emerging market has experienced significant volatility. These phenomena suggest that, despite its status as a major commodity exporter, Russia’s overall significance in the global economy may be limited. We can conclude that the shocks from COVID-19 are global and indiscriminate, affecting various markets uniformly. However, when the conflict starts, the impacts are primarily directed at commodities and Russia’s stock market, given its classification within MSCI Emerging Markets.

Table 1 gives descriptive statistics for all assets. It indicates that gas exhibits the highest average return and standard deviation. Additionally, the Jarque-Bera test rejects the hypothesis of normal distribution for the return series, while the ADF unit root test results show that all the series are stationary. Finally, we apply the Lagrange multiplier test to assess the presence of an ARCH effect. The results indicate that all sequences have an ARCH effect, suggesting the suitability of the GARCH model.

**Table 1:** Descriptive statistics of daily returns.

	Brent	Gas	Gold	Wheat	Developed	Emerging	Frontier
*Min* .	−12.41	−18.08	−5.86	−10.02	−10.44	−6.94	−12.69
*Max* .	7.50	12.45	4.97	8.28	8.41	5.57	2.36
*Mean*	0.05	0.10	0.03	0.06	0.03	−0.01	−0.01
*Std* . *dev* .	1.73	2.20	0.95	2.03	1.23	1.16	0.80
*JB* *test*	2251**	8652**	582**	149**	8980**	1250**	221529**
*ADF*	−11**	−10**	−11**	−9.3**	−9.1**	−9.5**	−7.5**
*ARCH*(20)	85**	277**	73**	278**	365**	259**	100**

Note: Std. Dev. represents standard deviation. JBtest is the Jarque-Bera test for the normal distribution. ADF represents Augmented Dickey-Fuller test of stationarity. ARCH(20) is the Autoregressive Conditional Heteroskedasticity model where the number 20 indicates the lag order. ∗∗ and ∗ denote p<0.01 and *p* < 0 . 1, respectively.

**Fig 4:**
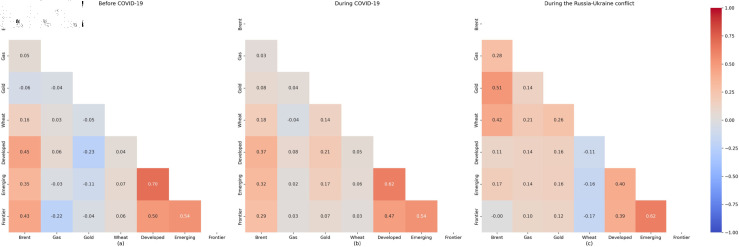
Heatmap of Pearson’s correlation matrix between oil, gas, wheat, gold, and the stock markets over the three analysed periods.

Fig 4 presents the Pearson correlation coefficients among oil, gas, wheat, gold, and the stock markets across three periods. It is evident that oil consistently positively influences the returns of all three selected stock markets. Before the outbreak of the Russian-Ukraine conflict, correlations between natural gas and the stock markets are mainly negligible or negative but significantly turn positive afterwards. Conversely, while correlations between wheat and stock indices returns remain consistently near zero, they shift to negative during the Russia-Ukraine conflict. Regarding gold, its correlations with the stock markets fluctuate from negative to positive following the onset of the conflict.

## 4 Results and discussion

This section summarizes the quantile coherency analysis of global equities and commodities across pre-COVID-19, COVID-19, and the Russia-Ukraine conflict. We examine how asset correlations shift across different market conditions and quantiles. And we also use the hedging efficiency method as a supplement to our analysis.

### 4.1 Empirical results of the quantile coherency approach

**Fig 5:**
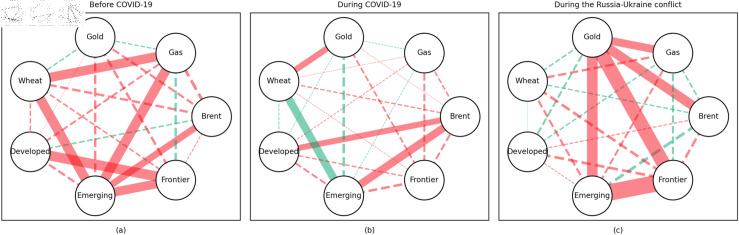
The 5th quantile coherency network in short term.

**Fig 6:**
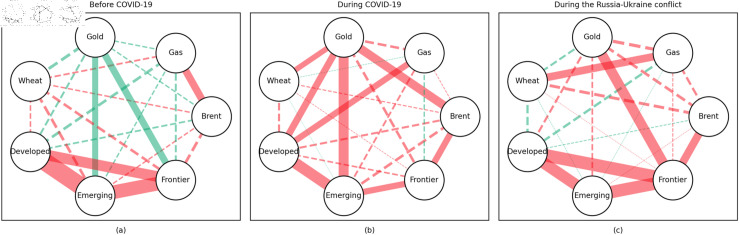
The 50th quantile coherency network in short term.

**Fig 7:**
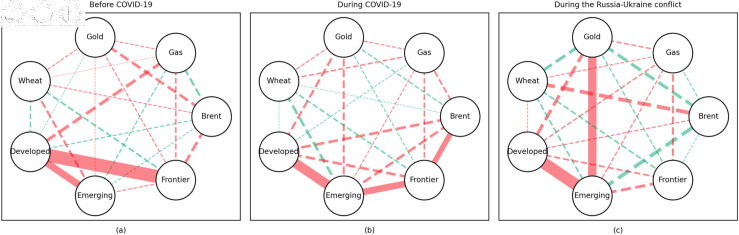
The 95th quantile coherency network in short term.

**Fig 8:**
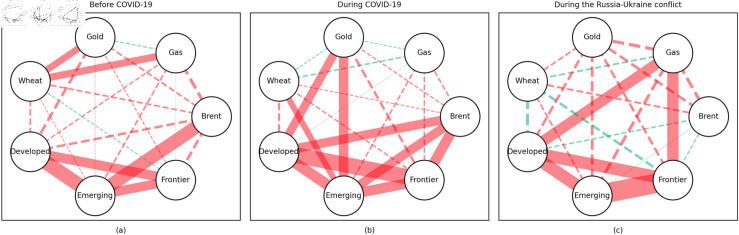
The 5th quantile coherency network in medium term.

**Fig 9:**
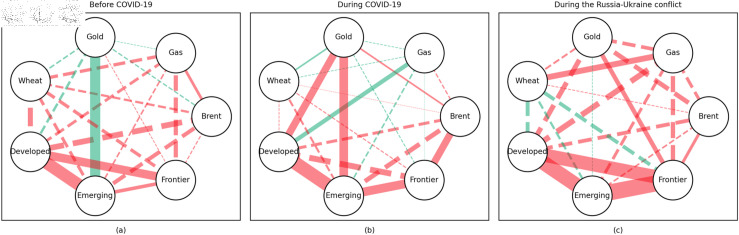
The 50th quantile coherency network in medium term.

**Fig 10:**
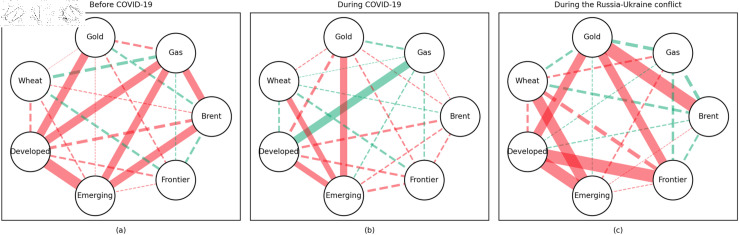
The 95th quantile coherency network in medium term.

**Fig 11:**
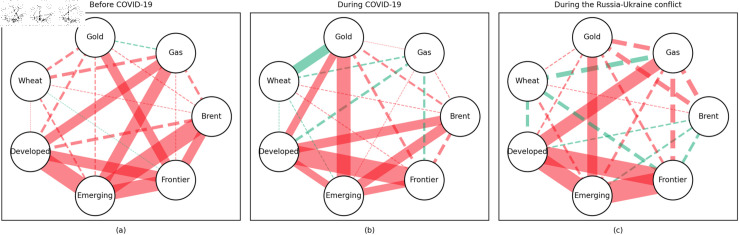
The 5th quantile coherency network in long term.

**Fig 12:**
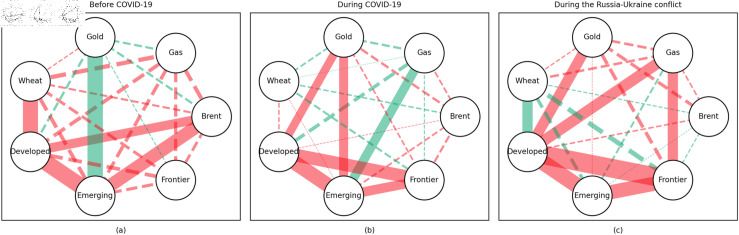
The 50th quantile coherency network in long term.

**Fig 13:**
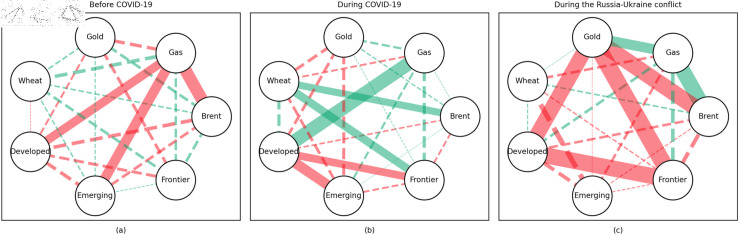
The 95th quantile coherency network in long term.

Fig 5 to 13 show the network structures of 0.05, 0.5 and 0.95 quantiles (representing extreme positive, normal and extreme negative return) coherency matrices in different frequency domains (one week, one month, and one year). The corresponding network structures of global equities and commodities are depicted for three distinct periods: before COVID-19, during COVID-19, and amid the Russia-Ukraine war. Note that a level of statistical significance of 0.1 is given in each QC matrix, with 0 set for insignificant values. The red (green) lines show each network’s positive (negative) quantile coherency. The solid (dotted) lines present significant (insignificant) tail dependencies. We analyze the differences in asset relationships under different quantile conditions within the same period. At the same time, we evaluate differences across different periods through vertical comparison. We consider the pre-pandemic period as representing normal conditions, while the periods during COVID-19 and the Russia-Ukraine war are viewed as particular states characterized by further shocks. During COVID-19, markets, including supply, demand, and logistics, are affected by all-around shocks. Conversely, supply shocks primarily impact markets during the war, particularly in energy and agricultural commodities.

#### Short-term quantile coherency results

At the 0.05 quantile (bear market): Before the pandemic, the energy market exhibited synchronized movements with the emerging stock markets, while gold demonstrated strong safe-haven properties during this period. During the pandemic, natural gas emerged as a stronger safe-haven asset compared to oil, with both gold and wheat also displaying safe-haven characteristics, particularly with wheat showing notable effectiveness. During the Russia-Ukraine conflict, the energy market showed no correlation with stock markets, and safe-haven assets stood out, although gold lost its safe-haven status.

At the 0.5 quantile (normal market): Before the pandemic, there was no significant correlation between the energy market and stock markets. However, during the pandemic and the Russia-Ukraine war, the correlation between oil and stock markets strengthened. During the Russia-Ukraine conflict, natural gas became an important hedging tool for stock markets, while wheat consistently served as a stable hedging asset across all periods. Notably, gold lost its hedging properties during both the pandemic and the Russia-Ukraine conflict, showing a positive correlation with stock markets.

At the 0.95 quantile (bull market): Across all three periods, there was no significant correlation between the energy market and stock markets. However, during the Russia-Ukraine conflict, gold exhibited a strong positive correlation with stock markets. Wheat, on the other hand, showed no significant connection with stock markets during this period.

#### Medium-term quantile coherency results

At the 0.05 quantile (bear market): Before and during the pandemic, natural gas served as a strong safe-haven asset, while oil showed a significant positive dependence on stock markets. However, during the Russia-Ukraine conflict, oil exhibited safe-haven properties. Gold and wheat were reliable safe-haven assets both before the pandemic and during the Russia-Ukraine conflict. Additionally, we observed a positive interdependence between gold and wheat before the pandemic, as confirmed by previous research.

At the 0.5 quantile (normal market): Natural gas consistently demonstrated good hedging properties across all three periods, particularly during the pandemic, where it showed a notable negative correlation with stock markets, reinforcing its effectiveness as a safe-haven asset. In contrast, oil did not display hedging attributes during the pandemic or the Russia-Ukraine conflict, with a weak correlation to stock markets. Gold failed to act as an effective hedging tool in both periods, while wheat showed robust hedging functionality during these times.

At the 0.95 quantile (bull market): Before the pandemic, oil and natural gas had some correlation with stock markets, but this relationship disappeared during the pandemic and the Russia-Ukraine conflict. Gold maintained a positive correlation with stock markets in all periods, while wheat showed some correlation with stock markets during both the pandemic and the Russia-Ukraine conflict.

#### Long-term quantile coherency results

At the 0.05 quantile (bear market): Oil showed a significant positive correlation with stock markets before and during COVID-19, while gas did not correlate with stock markets during the pandemic. Gold maintained a positive correlation with stock markets across all periods, whereas wheat consistently acted as a stable safe-haven asset for stock markets.

At the 0.5 quantile (normal market): Before the pandemic, there was no significant correlation between natural gas and stock markets. During the pandemic, natural gas exhibited negative dependence on stock markets, indicating strong hedging properties. Oil did not display any dependence on stock markets during the pandemic or the Russia-Ukraine conflict. Gold did not serve as a good hedging tool in either period, while wheat consistently acted as an effective hedging asset during both events.

At the 0.95 quantile (bull market): Natural gas did not show any significant positive correlation with stock markets during the pandemic or the Russia-Ukraine conflict. Oil had no clear connection with stock markets across all periods. Gold exhibited a positive correlation with stock markets only during the Russia-Ukraine conflict, while wheat showed no connection with stock markets across all periods.

In summary, in both the short and medium term, natural gas outperformed oil during the pandemic and the Russia-Ukraine conflict, particularly in bear and neutral market conditions, showing significant potential as a hedging tool [70,71]. This finding is further supported by the Pearson correlation results. In contrast, over the long term, oil outperformed natural gas. During the Russia-Ukraine conflict, oil exhibited safe-haven properties, consistent with the findings of previous studies [50,72,73]. The weak connection between food markets and other markets suggests that food commodities can act as safe havens during crises. On the other hand, the hedging characteristics of gold were inconsistent across different quantiles, particularly during the pandemic and the Russia-Ukraine conflict, where it tended to lose its hedging and safe-haven functions, contrary to the findings in [26,74,75]. During the pandemic, global transportation restrictions and the stagnation of logistics and human mobility severely disrupted supply and demand [76–80]. The pandemic significantly enhanced the correlation between financial markets, particularly under extreme negative shocks, where multiple markets became more interconnected, echoing findings from previous research [81]. At the 0.95 quantile (bull market), oil and natural gas showed weaker correlations with stock markets during the Russia-Ukraine conflict. Short-term network analysis revealed minimal strong connections between markets, indicating relatively loose inter-asset linkages under extremely positive market conditions.

**Fig 14:**
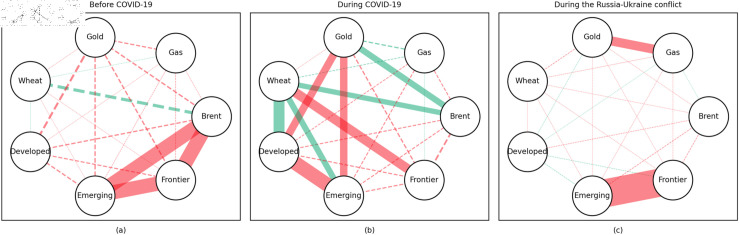
The 1th quantile coherency network in short term for robustness check.

**Fig 15:**
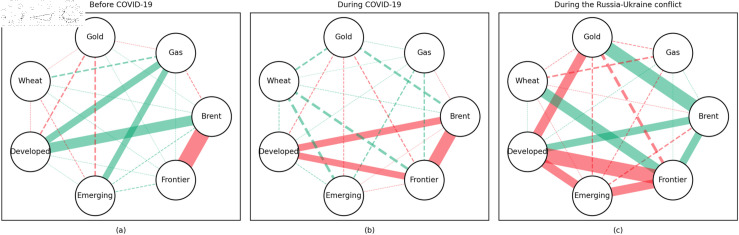
The 99th quantile coherency network in short term for robustness check.

**Fig 16:**
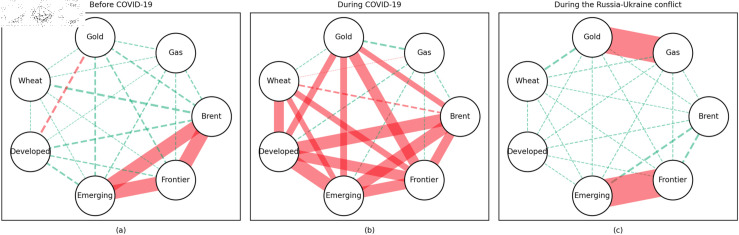
The 1th quantile coherency network in medium term for robustness check.

**Fig 17:**
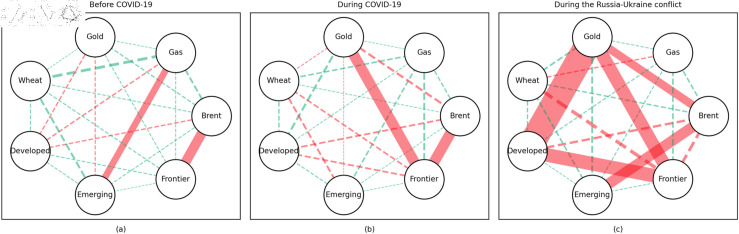
The 99th quantile coherency network in medium term for robustness check.

**Fig 18:**
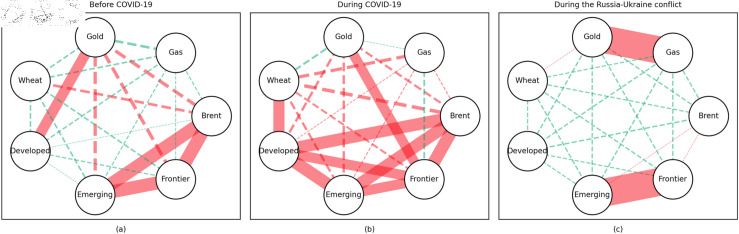
The 1th quantile coherency network in long term for robustness check.

**Fig 19:**
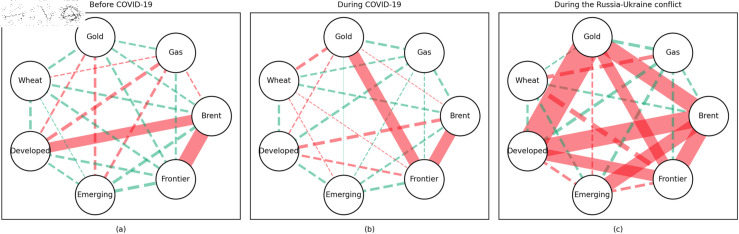
The 99th quantile coherency network in long term for robustness check.

In addition, the predominance of positive correlations among assets under the 0.05 quantile coherency confirms the literature’s viewpoint that asset returns tend to co-move in distress periods [82]. The analysis also uncovers asymmetric effects in the tail dependencies between stock and commodity futures markets [83–87]. Furthermore, it is noteworthy that the correlations among stock markets during bear markets remain similar across the three distinct periods. This consistency highlights the persistence of market behaviour during economic downturns, regardless of external shocks. For robustness, we use quantiles 0.01 and 0.99 to replace quantiles 0.05 and 0.95 as the very extremely low and extremely low returns quantiles, respectively. The results of the robustness test of the quantile coherency approach are shown in Fig 14 to 19. Evidently, there is no substantial change in the outcome.

### 4.2 Empirical results of hedging efficiency

**Fig 20:**
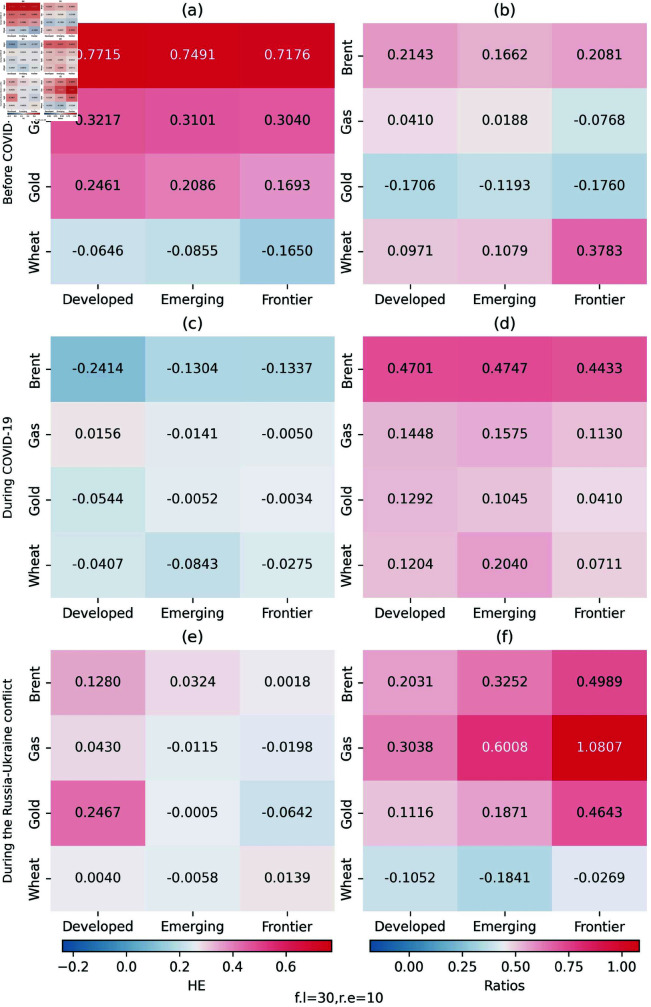
Hedging effectiveness (HE) indices and hedge ratios.

We estimate the hedging effectiveness (HE) index and the average hedging ratios (AHR) to reinforce the results of the quantile coherency approach. Given the sample size in this study, we construct 30 one-period-ahead forecasts and modify the model for every 10 observations. Note that the higher the HE, the better the effectiveness of an asset to hedge other assets i.e., the greater the risk reduction. The AHR shows the required hedging position of an asset to hedge other assets. If AHR is positive, the investors should hedge the stock market by taking an opposing position in brent (gas, gold or wheat) and vice versa. Fig 20 displays the results of the calculation. The empirical findings indicate that in the period of before COVID-19, the portfolios of stock indices had a high hedging effectiveness after adding brent, gas and gold. Among the three stock indices, the HE indices of the stock index of frontier markets are the smallest. The results verify our previous analysis to some extent, because we find the superiority of brent among all assets during the Russia-Ukraine conflict. The portfolios of stock indices during COVID-19 have an ineffective hedging effectiveness after adding these assets.

The average hedge ratio (AHR) informs us about the appropriate action—whether to purchase or sell an asset, such as brent, gas, gold or wheat—when adjusting our stock portfolio’s hedge. We can find that the majority of the hedge ratios are positive, suggesting that only a short position rather than a long one in an asset included in the portfolio of a long position in a stock market index can bring higher hedging benefits. The highest HR reaches 0.4701 when brent is used to hedge against developed stock markets during the COVID-19. This implies that a $1000 long position in the stocks of the developed stock market is hedged by taking a short position for $470.1 in the brent.

**Fig 21:**
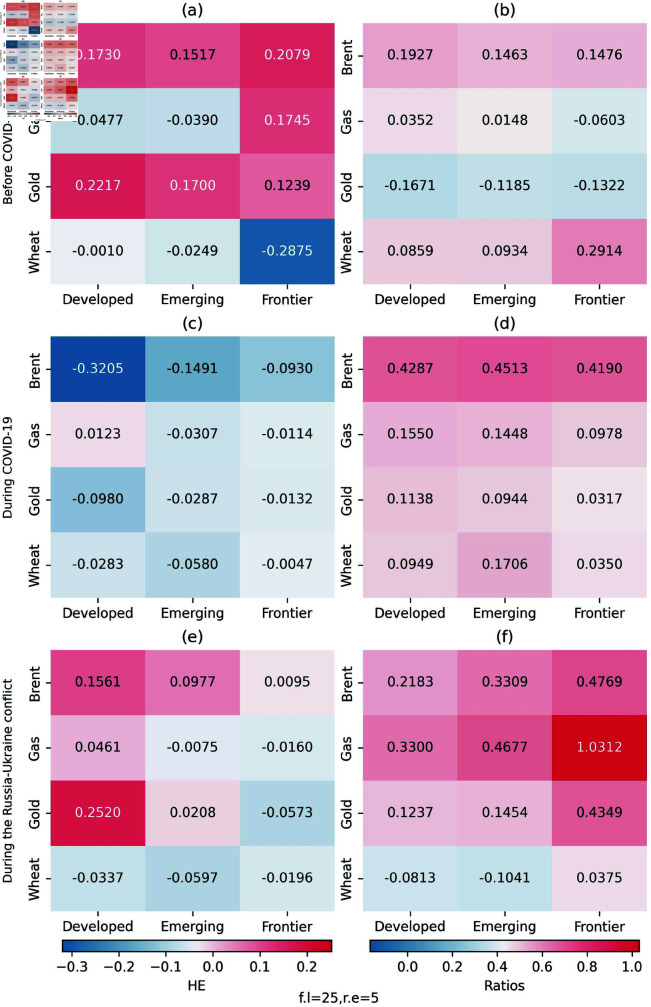
Hedging effectiveness (HE) indices and hedge ratios for robustness check.

We present the hedging effectiveness indices and hedge ratios computed by different number of model refits and the forecast length in Fig 21 as robust tests. The HE indices and hedge ratios are calculated from a fixed width rolling analysis which constructs 25 one-period-ahead forecasts and refits every 5 observations. Obviously, the results almost remain the same, which confirms the robustness of our original model.

### 4.3 Discussion

Based on the results of the above study, we find that: Firstly, the market performances of natural gas and oil vary significantly over different periods. In the short term, especially in bear and normal market conditions, natural gas outperforms oil. This means that during the downward or stable fluctuation stages of the market, natural gas, as a hedging tool, can more effectively diversify risks. One reason is that the natural gas supply is relatively stable and less directly impacted by short-term geopolitical conflicts. Moreover, during the period of accelerated energy structure transformation, the industrial and civilian demands for natural gas have a certain rigidity. In the long run, however, oil performs better, reflecting that oil’s fundamental and dominant position in the global energy system is hard to shake in the short term. The long-term industrial layout, large-scale infrastructure investment, and international energy trade system are still oil-centric and have strong value resilience in the long cycle. During the Russia-Ukraine conflict, the hedging property exhibited by oil shows that as the king of global commodities, any fluctuation in the price of oil has far-reaching consequences and the conflict led to supply panic, prompting investors to pour funds into the oil market for hedging.

Regarding the food market, its weak connection with other markets is of great significance. When crises rage, food commodities can stand alone and become a safe haven for investors. This is because grain, as a basic necessity for human survival, has extremely low demand elasticity. Whether the economy prospers or declines, people’s basic consumption of grain will not change substantially, thus ensuring the relative stability of its price and isolating it from the violent fluctuations of the financial market.

The gold market presents complex and contradictory characteristics. In traditional perception, gold is widely recognized as a safe-haven asset. However, this study found that during the pandemic and the Russia-Ukraine conflict, its hedging and safe-haven functions failed. This may be due to the high complexity and integration of modern financial markets. Under the impact of extreme crises, investors’ panic soared and the demand for capital liquidity increased sharply. Furthermore, during crises, central banks and governments around the world frequently introduced unconventional monetary policies and fiscal stimulus plans. These powerful intervention measures distorted the traditional market supply-demand relationship and risk preference patterns, breaking the hedging logic on which gold originally relied.

Moreover, during the pandemic, global transportation was blocked, logistics stagnated and people’s movement was restricted, which deeply disrupted the supply-demand balance. This not only directly impacted the production and delivery of related commodities but also triggered a chain reaction in the financial markets, causing the correlations among various financial markets to soar. However, in the bull market, during the Russia-Ukraine conflict, the correlations between oil, natural gas and the stock market weakened, revealing that during the stage of extreme optimism in the market and rapid rises in asset prices, the driving factors of the energy market and the stock market diverged. The energy market is dominated by geopolitics and physical supply and demand, while the stock market is driven by corporate earnings expectations and macroeconomic growth prospects, with different rhythms.

The hedging efficiency results show that before COVID-19, the addition of Brent crude oil, natural gas and gold to the portfolio of the stock index could improve the effectiveness of the hedging, reflecting that under normal market conditions, these assets could rely on their own characteristics to protect stock investments. The HE index of frontier market stock indices is the smallest, indicating that frontier markets themselves have high volatility, low maturity, and weak buffer capacity against external shocks, and thus require more diversified asset allocation to enhance stability. During the Russia-Ukraine conflict, Brent crude oil showed obvious advantages, because of its status as the benchmark of international oil prices and price fluctuations containing rich information and investment opportunities. In contrast, during COVID-19, the addition of these assets had poor hedging effects. The pandemic triggered a global economic shutdown and a sharp decline in demand, leaving both commodity and stock markets in trouble and disabling the coordinated hedging function between assets, preventing investment portfolios from effectively diversifying risks. The results of the effectiveness of hedging further support the previous study to some extent.

## 5 Conclusion

This study provides empirical evidence on the impact of the Ukraine-Russia war on the correlation between energy commodities and global stock markets by applying a quantile coherency approach. It includes a comparative analysis under various conditions. We divide the daily data spanning from January 3, 2019, to October 6, 2022, into three periods for this study, which examines two significant energy commodities (natural gas and oil), three major stock markets, along with precious metals (gold) and agricultural products (wheat).

In the short and medium term, natural gas has outperformed oil during the pandemic and the Russia-Ukraine conflict, demonstrating potential as a hedging tool, especially under bearish and neutral market conditions. In contrast, in the long run, oil outperforms natural gas. During the Russia-Ukraine conflict, oil served as an effective hedging instrument, consistent with the observations of [49,72,73]. Wheat shows weaker connections with stock markets, suggesting its potential as a safe haven or hedging tool during crises. In contrast, the safe-haven and hedging properties of gold are inconsistent across quantiles, especially during the pandemic and the Russia-Ukraine conflict, when gold tended to lose its hedging and safe-haven character. Additionally, lower return quantiles across all assets show greater interconnection than higher return quantiles, particularly during pandemic. This aligns with the idea that joint returns exhibit higher dependency during economic contractions compared to expansions [19,88]. In addition, we use the hedging effectiveness index for further analysis. These findings echo the results of the quantile coherency approach to some extent.

The insights from our study can help investors choose appropriate energy commodities for hedging against various types of shocks. For policymakers, these findings suggest that policy adjustments can enhance market stability by considering the interdependencies between energy and financial markets under different shock scenarios. Furthermore, adopting innovative strategies such as energy communities [89] can stimulate the development of the energy market, promoting environmental, social, and economic progress.

This study has limitations, such as focusing on only two representative energy commodities in assessing their impact on global stock markets and the stock markets not being categorized by countries. Future research can provide a more comprehensive understanding of the two crises by filling these research gaps.
